# Reply to Koplenig and Wolfer: Global language analyses must account for relationships, location, and unbalanced binary data

**DOI:** 10.1073/pnas.2518837122

**Published:** 2025-10-08

**Authors:** Xia Hua, Lindell Bromham

**Affiliations:** ^a^Mathematical Sciences Institute, Australian National University, Canberra, ACT 2601, Australia; ^b^Research School of Biology, Australian National University, Canberra, ACT 2601, Australia

Koplenig and Wolfer (KW) (1) fail to account for important features of language data: i) Typological data (e.g., grammatical features) are often binary, unbalanced (far fewer languages with feature than without), and unevenly distributed (clustered in languages and regions); ii) autocorrelation due to relationships and distance cannot be captured in family or regional-level analyses. We show that aspects of our analysis (2) designed to deal with i) do not alter the conclusions, but failure to account for ii) explains KW’s results.

All statistical methods have assumptions, but some are more clearly violated than others. Bayesian (BIC)’s assumption of Gaussian posterior parameter distribution is violated for unbalanced binary variables like Polysynthesis. Using a numerical method that does not make assumptions about data distribution (reversible-jump-MCMC, rjMCMC) (3), which increases computational time by orders of magnitude, strengthens our conclusions (Table 1). RjMCMC estimates larger posterior inclusion probability (PIP) for Small_Family than BIC but similar PIP to maximum likelihood approximation. We use PIP only to rank the importance of predictors (2). Setting an arbitrary PIP threshold (e.g., PIP > 0.5) is inappropriate because PIP is underestimated for unbalanced binary predictors correlated with important continuous predictors. Small_Family correlates with altitude and L1_population, which require smaller sample sizes to achieve similar statistical power (4).

**Table 1.** Coefficient mean, SE, and PIP of predictors for 375 Polysynthetic (Table S1 in ref. 2) and 428 Extended list (Table S2 in ref. 2), which are unevenly distributed (Figure 2 in ref. 2) and phylogenetically clustered (see SI Appendix, Tables S1 and S2 in ref. 2)

Predictor	Polysynthetic			Extended		
	Mean	S.E.	PIP	Mean	S.E.	PIP
Altitude	0.21	0.031	1.000	0.26	0.045	1.000
Area	0.09	0.107	0.442	0.02	0.051	0.118
Bordering languages	−0.09	0.109	0.466	−0.02	0.053	0.143
Bordering languages/km	−0.15	0.121	0.654	−0.26	0.064	0.968
L1 pop	−0.30	0.031	1.000	−0.30	0.026	1.000
Roughness	−0.01	0.028	0.128	−0.10	0.059	0.818
Small family	0.02	0.085	0.085	0.11	0.183	0.300
Vehicularity	−0.01	0.067	0.056	−0.01	0.073	0.066

We ran 10_6_ iterations split in 8 runs, each starting from a probit model including one of 8 predictors (discarding first 10_4_ samples) and the eigenvector selected by the general formula of MC with multimatrix extension suggested by ref. 1. RjMCMC estimates larger PIP for Small_Family than BIC, but similar PIP to Maximum Likelihood approximation (0.294 after subtracting lowest possible PIP = 0.5). Convergence check passed Brooks and Gelman test, Heidelberg-Welch test, and Geweke’s test. Effective sample size for all predictors is >2.9 × 10_4_.

Moran’s coefficient (MC) (5) with unjustified normalization factor (1) performs well for nonnormal distributions (6), selecting the same eigenvector with similar values to the formula KW suggested (1), and is computationally efficient for ~2 × 10^4^ eigenvectors. Residual from ref. 7 was used for ordinal probit model only for endangerment, because *spfilteR* does not have residual for categorical variables. MC-based selection is recommended for binary regression for Eigenvector Spatial Filtering (ESF) (8), used as an exemplar in *spfilteR* (9) and proven to be the best approach for linear models (10). To show why selection based on model-fit is inappropriate, we rerun ESF as KW suggest. Akaike information criterion (AIC)-based selection (9) selects 55 eigenvectors, yet residual autocorrelation remains significant. The large number of eigenvectors gives a high chance of separating the rare polysynthetic languages from others simply by chance. This separation problem in binary regression is evident in unreliable estimates, unrealistically high model fit and failure to converge (1).

**Fig. 1.**
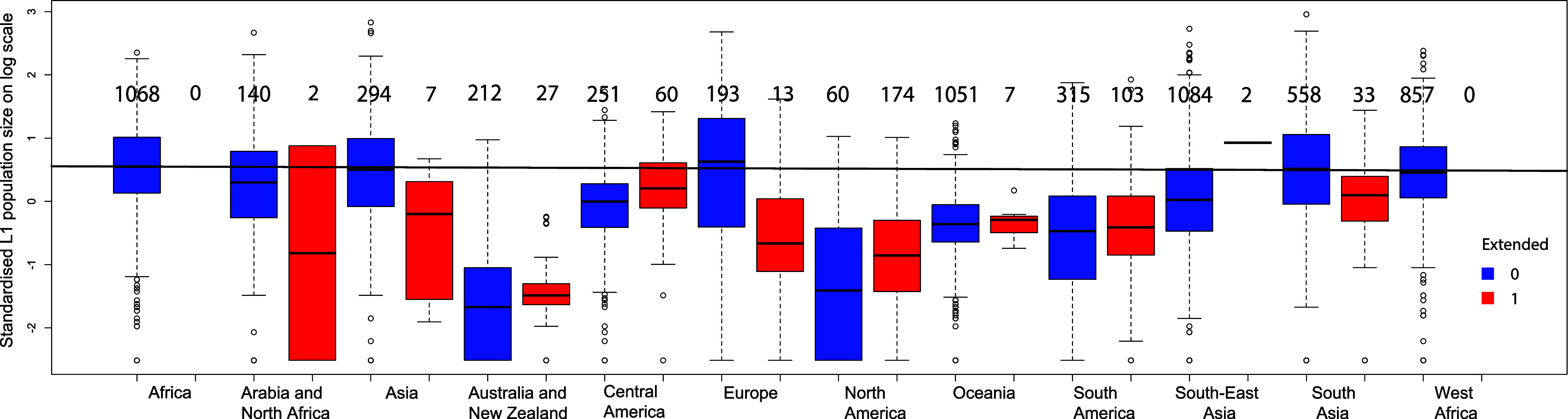
Population size of languages in each subregion. The number of polysynthetic languages (red) and nonpolysynthetic languages (blue) in each subregion is given on top of the corresponding boxplot for the “Extended list” (1). The line marks subregions with on average the largest population size, showing that subregion separates nonpolysynthetic languages in subregions with few or no polysynthetic languages from nonpolysynthetic languages in other regions, reducing information carried by the languages in these subregions to L1 population size. Uneven distribution of languages per subregion above or below the line indicates that heterogeneity in the occurrence probability of polysynthetic languages among subregions is not independent of population size, which violates an assumption of the logistic regression with mixed effects to account for autocorrelation.

KW’s counterintuitive results (1) are due to failure to account for autocorrelation. Using families and subregions (1) does not account for languages’ shared history and environments (e.g., assumes neighboring Polynesian languages are no more similar than they are to distantly related Taiwanese languages from the same family). The logistic mixed model violates the assumption that heterogeneity among groups is independent of predictors (Fig. 1): Subregions with predominantly nonpolysynthetic languages have larger populations, so subregion random effect separates the information carried by 69% nonpolysynthetic languages on the effect of L1_population. Adding family random effect worsens the separation problem: 35 families are all polysynthetic and 179 families have no polysynthetic languages, so the variance of family random effect is unrealistically large (~936) and model fit is unrealistically high (r^2^ > 99%) (1). The large contribution of random effects in ref. 1 is due to the separation problem, not because it better corrects for autocorrelation than ESF.
